# Supplementary material for: Attenuated Amplitude of Pattern Electroretinogram in Glaucoma Patients with Choroidal Parapapillary Microvasculature Dropout
Source: J Clin Med. 2022 Apr 28;11(9):2478. doi: 10.3390/jcm11092478 (PMC9101256; doi:10.3390/jcm11092478)

Figure S1. Relationship between angular width of MvD and N95 amplitude

In multivariate linear regression analysis, wider angular width was strongly associated with decreased N95 amplitude.

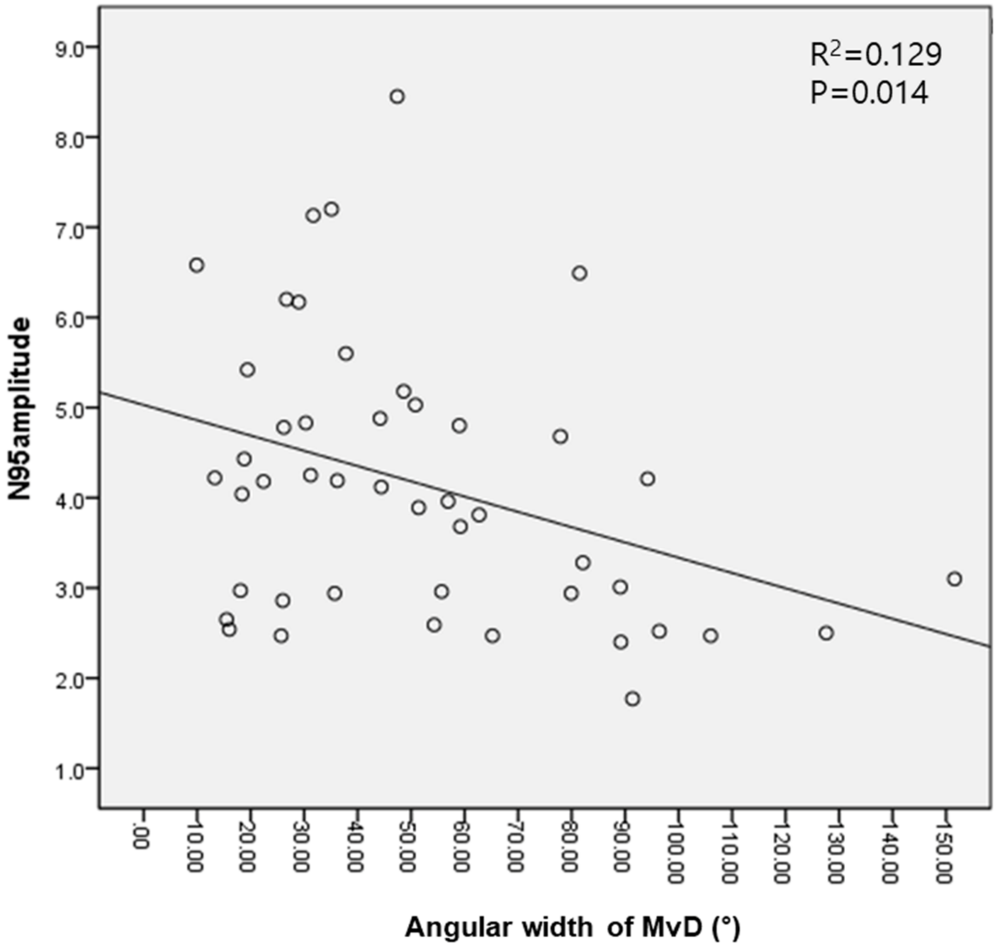

Supplement: Supplementary file 1 [file jcm-11-02478-s001.zip › jcm-1659413-supplementary.pdf]
